# Elevated NLR and PCT levels and reduced GCS score predict 90-day mortality in heatstroke: findings from a 13-year retrospective cohort study

**DOI:** 10.3389/fmed.2025.1599592

**Published:** 2025-06-27

**Authors:** Huili Guo, Jingjing Ji, Leifang Ouyang, Conglin Wang, Jinxin Jia, Zhifeng Liu

**Affiliations:** ^1^Department of Graduate School, Guangzhou University of Chinese Medicine, Guangzhou, China; ^2^Department of Medical Intensive Care Unit, General Hospital of Southern Theater of PLA, Guangzhou, China; ^3^Guangdong Branch Center, National Clinical Research Center for Geriatric Diseases, Guangzhou, China

**Keywords:** heatstroke, immune disorder, neutrophil to lymphocyte ratio, PCT, GCS

## Abstract

**Background and objective:**

Heatstroke is the most severe heat-related illness and is associated with high mortality rate. Inflammation and immune dysfunction are considered the key pathophysiological processes of heatstroke. The neutrophil-to-lymphocyte ratio (NLR) can reflect the states of innate and adaptive immune systems. The aim of the present study was to explore the predictive role of the NLR in heatstroke patients.

**Methods:**

This single-center retrospective cohort study included all patients with exertional-heatstroke (EHS) admitted to the intensive-care-unit (ICU) of the General Hospital of Southern Theater Command of PLA from June 2009 to May 2022. The dynamic changes in the main immune cell counts and ratios were recorded.

**Results:**

A total of 232 patients were enrolled. Survivors had decreased NLRs 24 h after admission, while nonsurvivors had continuously increased NLRs after admission. The AUC for the 24-h NLR was 0.928, with a cutoff of 11.981. The patients were divided into NLR-high (NLR > 11) and NLR-low (NLR ≤ 11) groups based on their 24-h NLRs. Patients in the NLR-high group had increased 90-day mortality. According to the multivariate analysis, an increased PCT level and decreased GCS score were independent risk factors for death in heatstroke patients with an NLR over 11, with odds ratios of 1.0999 (95% CI: 1.0050–1.2038, *p* value: 0.03863) and 0.6836 (95% CI: 0.5246–0.8908, *p* value: 0.00486), respectively.

**Conclusion:**

An NLR greater than 11 in the early phase could be an independent predictor of prognosis in heatstroke patients, and an increased PCT level and decreased GCS score were risk factors for a poor prognosis.

## 1 Introduction

In the past several years, heat waves have worsened due to climate change and its effects. High prevailing temperatures increase the risk of mortality for a diverse set of causes of death. Heat-related diseases are associated with increased morbidity, among which heatstroke is the most severe condition (1). Heatstroke is defined as a heat-related illness characterized by a rapid increase in core body temperature above 40°C and central nervous system dysfunction, including seizures, syncope, and coma (2). For heatstroke patients, the mortality rate can reach 63.2% in the intensive care unit. Data from the United States have shown that the median hospitalization cost was $17,372, which was a heavy social and economic burden on individuals, families, communities and countries (3).

Increased core temperature is a typical characteristic of heatstroke. Heat stress can directly induce damage to host cells, proteins, lipids and nucleic acids, potentially culminating in multiorgan damage and death (2). At present, the pathophysiological process of heatstroke is described as “sepsis-like”; that is, the key mechanism in the progression of heatstroke is immune disorder, including the overactivation of the innate immune system and the immunosuppression of the adaptive immune system (4). The overactivation of the innate immune system manifests as its functional activation and an increase in the neutrophil count. The immunosuppression of the adaptive immune system manifests as its functional suppression and a decrease in the lymphocyte count. In previous study, the neutrophil count was used to evaluate the immune response. However, for those patients with hypoimmunity or insufficient bone marrow mobilization, their neutrophil counts could be normal, and further analysis of neutrophil functions was limited due to the test equipment. Currently, researchers have shown that the neutrophil-to-lymphocyte ratio (NLR) can reflect the states of the innate immune system and adaptive immune system (5). Even in patents with hypoimmune status, the NLR can easily and quickly predict patient prognosis. As an immune indicator, the NLR has recently been found to have good predictive value for the severity of many diseases, such as sepsis, cancer, and inflammatory bowel disease. The early indication of a poor prognosis is beneficial for early intervention and the prevention of disease progression. Hence, the aim of the present study was to explore the role of the NLR in predicting heatstroke prognosis.

## 2 Methods

### 2.1 Study patients

For the present study, data were retrospectively collected from patients who were diagnosed with heatstroke and admitted to the intensive care unit of the General Hospital of Southern Theater Command in China from June 2009 to May 2022. The study was approved by the Research Ethics Commission of the General Hospital of Southern Theater Command of the PLA. The requirement to obtain individual informed consent for this retrospective analysis was waived.

### 2.2 Patient selection

Patients who met the following criteria were eligible for inclusion: (i) were aged ≥18 years; (ii) admitted to the hospital within 3 days of onset; (iii) had a history of strenuous activity or exposure to hot and humid weather; and (iv) had concurrent hyperthermia (central temperature above 40°C) and neurological dysfunction, including delirium, cognitive disorders, and disturbed consciousness. The exclusion criteria were as follows: (1) existing irreversible underlying diseases affecting mortality; (2) the presence of active or uncontrolled infection; (3) pregnancy or breastfeeding; (4) active malignancy; (5) the presence of hematologic disorders; (6) taking immunosuppressants or have a history of taking immunosuppressants in the past 1 month; and (7) the presence of a known hereditary immunodeficiency disorder.

### 2.3 Data collection and outcomes

The characteristics, organ function parameters and 90-day outcomes of the enrolled patients were collected. The dynamic changes in immune cell counts and ratios, including those of WBCs, neutrophils, monocytes, and lymphocytes, were recorded. The time points included admission and 24, 48, 72 h, 5, 7, and 14 days after admission. The NLR was calculated as the neutrophil count/lymphocyte count. The main outcome was 90-day mortality. The length of ICU stay was the secondary outcomes.

### 2.4 Statistical analysis

Since most continuous variables did not show a Gaussian distribution, the continuous variables are presented as the median and interquartile range and were compared with the Wilcoxon rank-sum test. Kaplan–Meier survival curves and the log-rank test were used. ROC curve analysis was used to evaluate the effects of the NLR on 90-day mortality in patients with heatstroke. To determine the independent risk factors for 90-day mortality in patients with severe heatstroke with increased NLRs, a Cox proportional hazards model was used. Significant indicators were identified using single-factor analysis, and those with *p* values < 0.1 were included in the multivariate Cox regression model. Odds ratios (ORs) and 95% confidence intervals (95% CIs) are presented. Statistical analysis was performed using R version 3.4.0. And it is an open-source software developed by its core development team, managed by the R Foundation, and registered in Vienna, Austria, Europe. *p* values (two-tailed) less than 0.05 were considered to indicate statistical significance.

### 2.5 Patient and public involvement

Patients or the public were not involved in the design, or conduct, or reporting, or dissemination plans of our research.

## 3 Results

### 3.1 Nonsurvivors showed a continuously increased NLR

Data from 232 patients with heatstroke were collected. All the patients were male, had a median age of 21 years, and had no underlying disease before heatstroke onset. During hospitalization, 90 patients experienced acute liver injury, 111 experienced acute kidney injury, and 100 experienced rhabdomyolysis. The final causes of death included direct brain injury, early shock, disseminated intravascular coagulation (DIC), septic shock due to later infection, and multiorgan failure. The dynamic changes in the white blood cell count, neutrophil count, lymphocyte count and NLR are shown in Supplementary Table S1. At admission, most patients showed increased white blood cell and neutrophil counts, while the lymphocyte counts of nonsurvivors were decreased at admission. The NLRs of both survivors and nonsurvivors were increased at heatstroke onset. Survivors had decreased NLRs 24 h after admission, while nonsurvivors had continuously increased NLRs after admission, especially at 24 and 72 h (Supplementary Table S1).

**Figure 1:**
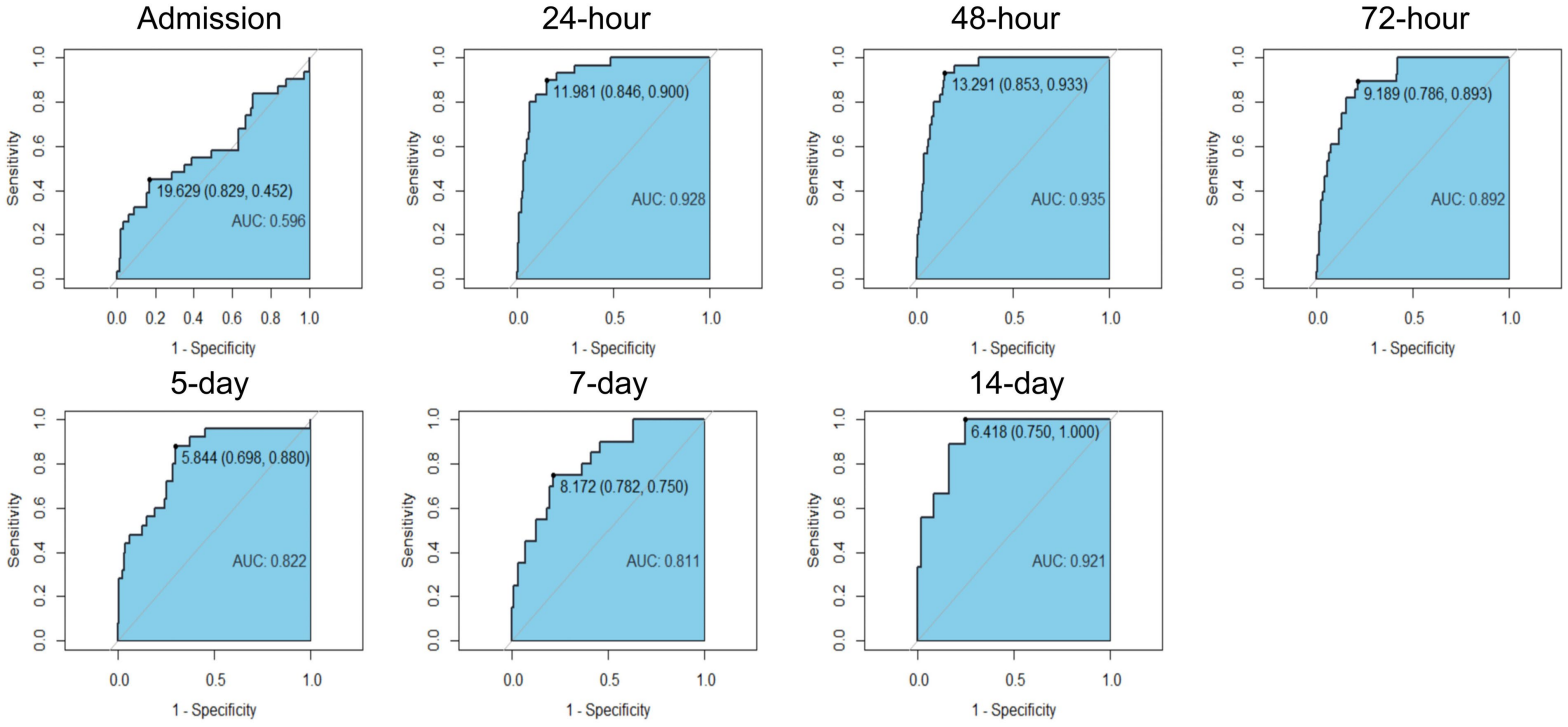
Receiver operating characteristic curves of neutrophil to lymphocyte ratio and at different time after admission.

Heatstroke patients exhibit overactivated innate immune responses and depressed adaptive immune responses (4). Since the NLR reflects the activity of both of these systems, we further calculated the area under the curve (AUC) for the NLRs. An AUC between 0.5 and 0.7 indicates that the diagnostic accuracy for diseases is low, an AUC between 0.7 and 0.9 indicates that the diagnostic accuracy is acceptable, and an AUC above 0.9 indicates that the diagnostic accuracy is high. In the present study, we found that the AUCs for 24-h, 48-h and 14-day survival were greater than 0.9 (Figure 1). The AUC for the NLR at 24 h was 0.928, with a cutoff of 11.981, the AUC for the NLR at 48 h was 0.935, with a cutoff of 13.291, and the AUC for the NLR at 14-day was 0.921, with a cutoff of 6.418 (Supplementary Table S2). The AUCs for 24-h, 48-h and 14-day AUCs were greater than 0.9. Since we wanted to find early markers, we decided to choose between 24 h after admission and 48 h after admission. The comparison of 24- and 48-h survival times using the DeLong method revealed no difference in prognosis (*p* = 0.413). Hence, clinically, their prognostic value is similar. To obtain an early warning indicator, we chose the 24-h NLR as the indicator, which could provide early warning for clinicians to recognize and intervene in these high-risk patients and further help block the progression of the disturbance of the inflammatory immune response.

### 3.2 Heatstroke patients with an NLR greater than 11 at 24 h after admission showed severe organ dysfunction

Our cutoff for the NLR at 24 h was 11.981. According to published studies, an NLR greater than 11 is considered high-grade inflammation (6, 7). The patients were divided into NLR-high (NLR > 11) and NLR-low (NLR ≤ 11) groups based on their 24-h NLRs. Seven patients were excluded due to data loss. Therefore, 225 patients were divided into the NLR-low group (*n* = 158) and NLR-high group (*n* = 67). Compared with patients in the NLR-low group, patients in the NLR-high group had increased APACHE II and SOFA scores.

**Figure 2:**
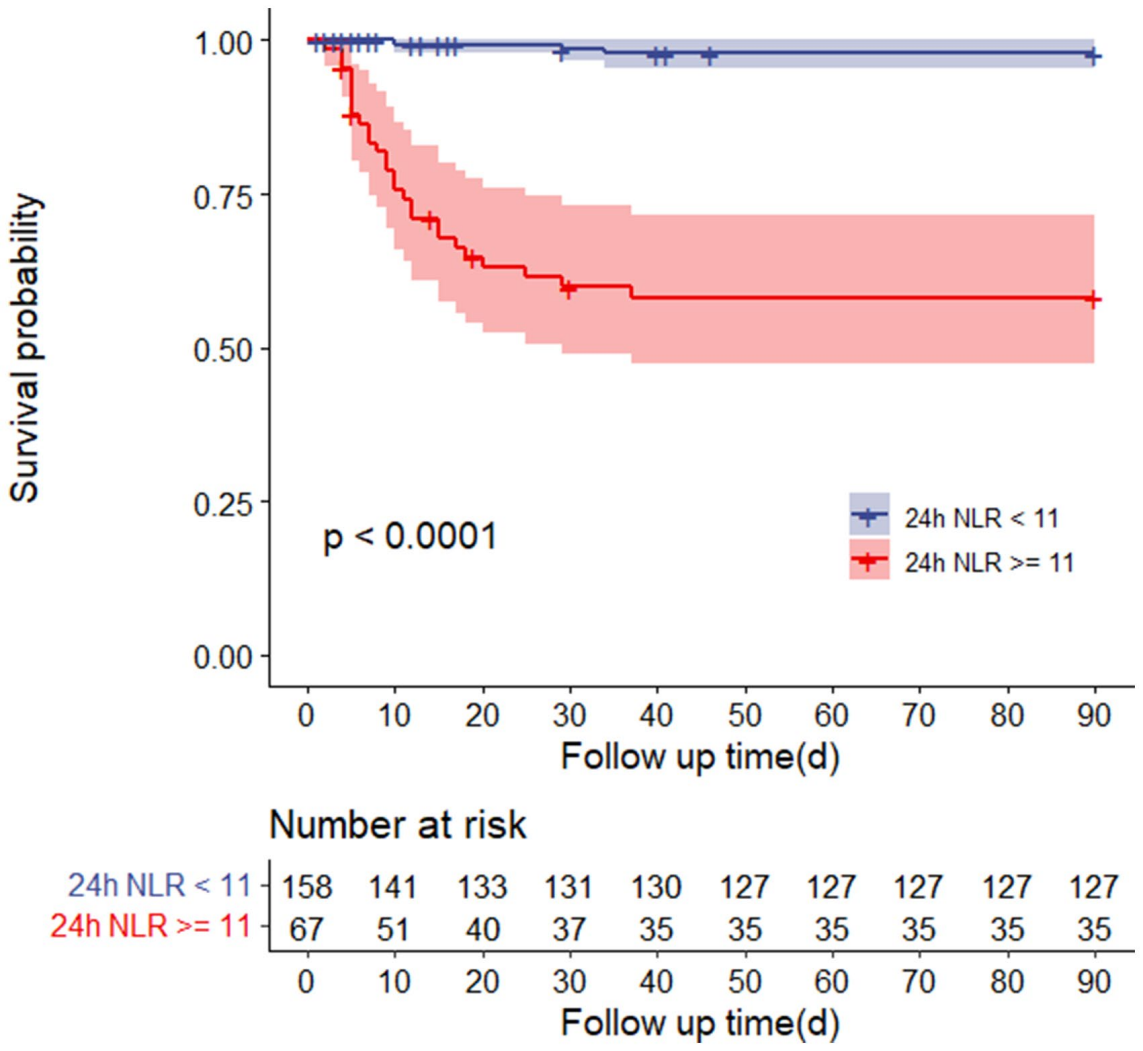
Survival curves of 90-day mortality rate in heatstroke patients with or without NLR over 11 after 24-h admission.

The organ dysfunction indices, including indices of hepatic function (TBIL, ALT, AST), kidney function (urea nitrogen, serum creatinine), rhabdomyolysis (CK, Mb), cardiac injury (CTNI), and coagulation function (PT, APTT, INR, TT, D-dimer), were increased in the NLR-high group. Patients in the NLR-high group also had a decreased GCS score (Table 1). In addition, patients in the NLR-high group had increased ICU length of stay and 90-day mortality (Supplementary Table S3). The 90-day mortality was 40.3% (27 in 67) for patients in the NLR-high group and 1.9% (3 in 158) for patients without an increase in the NLR. The survival time of EHS patients with an NLR greater than 11 was significantly shorter than that of patients with an NLR less than 11 (Figure 2).

**Table 1:** Comparison of the characteristics between the heatstroke patients in NLR low group and NLR high group.

Characteristics	Overall	NLR = < 11	NLR > 11	*P*-value
N	225	158	67	
APACHE II	2.00 [0.00, 4.00]	2.00 [0.00, 3.00]	9.50 [4.00, 17.25]	<0.001
SOFA	1.00 [0.00, 3.00]	1.00 [0.00, 2.00]	6.00 [3.00, 11.00]	<0.001
GCS	14.00 [13.00, 15.00]	14.00 [14.00, 15.00]	11.00 [6.00, 14.00]	<0.001
PLT	125.00 [57.00, 177.00]	155.00 [99.25, 191.75]	49.00 [31.00, 63.00]	<0.001
TBIL	26.80 [15.30, 55.30]	20.35 [13.55, 36.38]	57.70 [40.20, 103.00]	<0.001
ALT	233.00 [54.00, 928.50]	145.50 [32.50, 526.50]	1074.00 [270.00, 2088.00]	<0.001
AST	208.00 [60.00, 732.00]	114.00 [40.50, 416.00]	832.50 [242.50, 2693.75]	<0.001
BUN	6.10 [4.20, 7.90]	5.80 [4.10, 7.40]	7.30 [4.80, 9.60]	0.009
SCR	94.00 [75.00, 131.00]	88.00 [73.00, 110.00]	140.00 [93.00, 225.00]	<0.001
CysC	0.88 [0.78, 1.10]	0.85 [0.76, 0.96]	1.30 [0.88, 1.92]	<0.001
CK	1234.00 [377.00, 3425.00]	830.50 [245.00, 2738.50]	3015.00 [1057.00, 7304.00]	<0.001
CKMB	42.00 [23.00, 90.00]	32.00 [18.50, 65.00]	93.00 [38.50, 175.00]	<0.001
PT	15.60 [13.93, 19.92]	14.90 [13.70, 17.02]	23.30 [16.60, 29.20]	<0.001
INR	1.28 [1.08, 1.69]	1.17 [1.05, 1.41]	2.12 [1.34, 2.88]	<0.001
APTT	42.00 [38.32, 46.55]	40.35 [37.70, 44.23]	47.10 [41.78, 62.90]	<0.001
TT	17.20 [16.10, 19.17]	16.80 [15.90, 18.00]	21.80 [17.10, 25.92]	<0.001
Fib	2.60 [2.20, 3.30]	2.70 [2.20, 3.32]	2.60 [2.02, 3.27]	0.263
D. D	1.93 [0.60, 9.03]	0.95 [0.43, 2.56]	10.00 [6.56, 17.22]	<0.001
Mb	149.50 [48.94, 584.10]	106.70 [29.91, 259.20]	836.00 [237.00, 1000.00]	<0.001
CTNI	60.00 [10.00, 240.80]	23.06 [10.00, 91.20]	260.00 [62.00, 688.85]	<0.001
PCT	1.98 [0.86, 4.12]	1.37 [0.67, 2.96]	3.50 [1.81, 6.47]	<0.001
CRP	3.44 [3.03, 8.60]	3.44 [2.92, 6.28]	4.85 [3.30, 13.08]	0.11

ALT, alanine transaminase; APACHE-II, Acute Physiology and Chronic Health Evaluation-II; APTT, activated partial thromboplasting time; AST, aspertate aminotransferase; BUN, blood urea nitrogen; CK, creatine kinase; CKMB, creatine kinase-myocardial band; CRP, C-reactive protein; CTNI, cardiac troponin I; CysC, cystatin C; D. D, d-dimer; Fib, fibrinogen; GCS, Glasgow coma scale; INR, international normalized ratio; Mb, myoglobin; PCT, procalcitonin; PLT, Platelet; PT, prothrombin time; SCR, serum creatinine; SOFA, sequential organ failure assessment; TBIL, total bilirubin; TT, thrombin time.

### 3.3 An increased PCT level and decreased GCS score were risk factors for 90-day mortality in heatstroke patients with NLRs greater than 11 at 24 h after admission

To further determine the risk factors for death in patients with an increased NLR, we compared the organ function indices of the survivors and nonsurvivors (Supplementary Table S4). The nonsurvivors had more severe disease than did the survivors, as evidenced by a decreased GCS score and increased TBIL, AST, SCR, CysC, PT, INR, APTT, TT, D-dimer, Mb, CTNI, PCT and CRP levels. Since the number of variants in the multivariant analysis should not exceed 1/10, in the present study, the number of variants was not greater than 6. Therefore, the functional indices for each system were chosen for the multivariant analysis, that is, TBIL, SCR, APTT, CTNI, PCT and GCS score (Table 2). According to the multivariate analysis, an increased PCT level and decreased GCS score were found to be independent risk factors for death in heatstroke patients with NLRs greater than 11, with odds ratios of 1.0999 (95% CI: 1.0050–1.2038, *p* = 0.3863) and 0.6836 (95% CI: 0.5246–0.8908, *p* = 0.00486), respectively.

**Table 2:** Risk factors for the heatstroke patients with NLR over 11.

Characteristics	HR (95% CI)	*P*-value
SCR	1.0068 (0.9956, 1.0181)	0.23273
APTT	1.0067 (0.9899, 1.0237)	0.43833
CTNI	1.0007 (0.9997, 1.0017)	0.15803
PCT	1.0999 (1.0050, 1.2038)	0.03863
GCS	0.6836 (0.5246, 0.8908)	0.00486
TBIL	0.9927 (0.9833, 1.0023)	0.13609

APTT, activated partial thromboplasting time; CTNI, cardiac troponin I; GCS, Glasgow coma scale; PCT, procalcitonin; SCR, serum creatinine; TBIL, total bilirubin.

## 4 Discussion

The present retrospective cohort study showed that a high NLR greater than 11 in the early phase could be an independent predictor of prognosis in heatstroke patients. Severe organ dysfunction was detected in heatstroke patients with an NLR greater than 11 at 24 h after admission, and an increased PCT level and decreased GCS score were risk factors for these patients.

Our results showed that from admission to 14 days after admission, the AUC of NLR in heat stroke patients first increased, then decreased, and finally increased. (1) From admission to 48 h post-admission, the AUC of NLR showed continuous elevation. This was attributed to: (i) excessive neutrophil activation during the acute phase, where direct fever stimulation activated Toll-like receptors, promoting inflammatory cytokine release from neutrophils and leading to a “cytokine storm”; (ii) lymphocytopenia resulting from elevated stress-induced glucocorticoids and increased apoptosis, causing immunosuppression. This period represents a critical therapeutic window requiring aggressive cooling, anti-inflammatory, and immunomodulatory interventions. (2) From 72 h to 5 days post-admission, the AUC of NLR demonstrated significant decline, potentially due to therapeutic interventions and immune exhaustion. Treatment measures reduced heat stress injury and attenuated neutrophil activation. Meanwhile, lymphocytes remained at persistently low levels due to unresolved apoptosis or bone marrow suppression, collectively contributing to decreased AUC. During this phase, vigilance against immunosuppression and infection prevention are warranted. (3) From 7 to 14 days post-admission, the AUC of NLR rebounded, likely reflecting immune reconstitution and secondary infections. Lymphocyte counts gradually recovered during immune reconstruction while residual inflammation persisted. Additionally, opportunistic infections following prolonged immunosuppression could trigger secondary neutrophil elevation. This phase necessitates infection screening and immune recovery assessment. In conclusion, dynamic monitoring of AUC of NLR from admission to 14 days post-admission holds significant clinical value in managing heatstroke patients.

An increase in core temperature is a characteristic of heat stroke, and it is the subsequent cause of multiorgan damage. In this study, heatstroke patients with an NLR greater than 11 at 24 h after admission showed severe organ dysfunction. The organ dysfunction indices, including indices of hepatic function (TBIL, ALT, AST), kidney function (urea nitrogen, serum creatinine), rhabdomyolysis (CK, Mb), cardiac injury (CTNI), and coagulation function (PT, APTT, INR, TT, D-dimer), were increased in the NLR-high group. Patients in the NLR-high group also had a decreased GCS score. Recent studies have shown that severe heatstroke is caused by dysfunctional heat regulation accompanied by an acute phase response and changes in the expression of heat shock proteins, and the subsequent multiorgan injury is caused by the complex interaction between the cytotoxic effects of heat and the host inflammatory and coagulation responses. The pathophysiological process of heat stroke is considered to be “sepsis-like,” manifesting as a proinflammatory cytokine storm in the circulation and a change in the number and proportion of immune cells. In heatstroke patients, DuBose et al. reported that after exercise, the number of granulocytes, monocytes, and lymphocytes in the circulation increased, and in exertional heatstroke patients, the total number of leukocytes increased significantly, but mainly the number of granulocytes and monocytes, and the number of T lymphocytes decreased (8), suggesting that immune cells are involved in the process of heatstroke. The NLR was more effective at reflecting the state of the innate immune system and specific immune system than were the neutrophil count and lymphocyte count. The NLR has been used to predict the severity of sepsis, caner, and inflammatory bowel disease (9, 10). In the present study, we are the first to report that an NLR greater than 11 in the early phase could be an indicator of poor prognosis in heatstroke patients. The determination of this index is easy and inexpensive, helping clinicians recognize patients at high risk and provide early intervention. Furthermore, it can also be utilized as an evaluation criterion in future developmental therapeutic intervention studies.

Our results also showed that heatstroke patients with increased NLRs had more severe organ damage. Immunological derangement and organ damage form a vicious cycle in heatstroke. On the one hand, heat stress can induce parenchymal cell death (11, 12), while heat stress usually induces the activation of interstitial cells, such as fibroblasts and Kupfer cells (13, 14). These interstitial cells exhibit proinflammatory properties after heat stress, which further induces the release of chemoattractants and the recruitment of immune cells. The recruited immune cells, especially neutrophils, can release more proinflammatory cytokines, aggravating organ dysfunction (15). On the other hand, innate immune cells can be activated in the early stage after heat stress. Proinflammatory cytokines and chemokines from activated immune cells result in massive amplification of the inflammatory process. Heat stress can increase the adhesive capacity of neutrophils, and interactions between neutrophils and endothelial cells are increased (16). Neutrophils are exuded to the tissue interstices, leading to organ injury (17). A previous study revealed that under appropriate heat stress conditions, the lymphocyte count increases. However, when the degree of heat stress increases above the threshold value, the lymphocyte count decreases significantly (18). The mechanism involved in the heat-induced decrease in lymphocyte count is still unclear. One explanation is that heat stress can activate apoptosis signaling in lymphocytes (19). Some researchers speculate that lymphocyte development and maturation are limited under heat stress conditions (20). A decrease in lymphocytes, especially Treg cells, is related to organ dysfunction in heatstroke mice (21). In addition, overactivated neutrophils can release death signals, such as TNF-*α*, which can lead to lymphocyte apoptosis. Due to these potential interactions, the NLR was more valuable in the prediction of immune disorders under heat stress.

We also found that an increased PCT level and decreased GCS score were risk factors for heatstroke patients with an NLR greater than 11 in the early phase. Under physiological conditions, PCT is mainly secreted by the parathyroid gland, while in the inflammatory state, many tissues throughout the body can secrete PCT. Currently, the PCT level has been used as a marker for systemic inflammation. In heatstroke patients, an increased PCT level indicated that they were in a much more severe inflammatory state. In addition, the PCT level is a valuable predictor for infection, especially for gram-negative bacterial infections (22). Hence, for patients with an increased NLR, the risk of death increased when the NLR was combined with infection. This finding might be due to the decrease in lymphocytes. Patients with a hypoimmune status, insufficient humoral immunity and insufficient cellular immunity showed a decreased ability to eliminate the pathogen. Heat stress results in intestinal epithelial damage, increased permeability, and intestinal barrier dysfunction. Endotoxin translocation from the intestine is increased (23). Therefore, it is crucial to implement gastrointestinal protective measures for heat stroke patients with elevated NLRs, such as the use of gastric mucosal protective agents, gastrointestinal motility regulators, and rational administration of antibiotics for immunomodulatory therapy, which may contribute to a more favorable prognosis.

In heatstroke patients with a high NLR, another independent risk factor was the GCS score. Central nervous system disorders are a specific characteristic of heatstroke, and brain damage is related to the prognosis of heatstroke patients, both in the short-term and long-term. One explanation is that neurons are sensitive to heat, and heat stress can directly lead to neuronal cell death and further neurological dysfunction (24). Another explanation is neuroinflammation. Due to the presence of the blood–brain barrier, it is still unclear whether the degree of intracranial inflammation affects the inflammatory response in the circulation during heatstroke. Thus, immune cells in the central nervous system are activated (25). Heat stress can promote the M1 polarization of glial cells, immune cells in the central nervous system, while the proportion of M2 glial cells, which are capable of phagocytosing necrotic cells and pathogens, decreases (26). In addition, the nervous system plays an important role in regulating immune function. Moreover, heatstroke-induced neurological impairment might exacerbate systemic immune disorders, leading to an increased risk of poor prognosis.

This study has several limitations. Firstly, it is a single-center ICU retrospective cohort study that only included patients with severe heat stroke, which introduces geographical and population limitations. As a retrospective cohort study, it also carries a certain risk of selection bias that may affect the reliability of the results. Future prospective studies are needed for further validation. Secondly, although the study excluded some underlying diseases that affect mortality, there is still the possibility of other confounding factors. We have identified NLR > 11 as an independent predictor of prognosis in heat stroke patients, but it remains unclear whether this threshold can be applied to other types of heat-related illnesses, such as febrile seizures or heat exhaustion. Additionally, the current study has established a correlation based on the propensity of clinical data but has not established a causal link. Further research is needed to clarify whether neutrophils and lymphocytes directly influence the process of 90-day mortality. In summary, the current level of evidence is limited, and more clinical studies are required to determine the optimal cutoff value for NLR and to validate its utility in the prognostic assessment of severe heat stroke patients. Further exploration is also needed to assess the value of NLR-based risk stratification in guiding treatment decisions.

## 5 Conclusion

An NLR greater than 11 in the early phase could be an independent predictor of prognosis in heatstroke patients. Early-stage heatstroke patients with an NLR greater than 11 had severe organ dysfunction, and an increased PCT level and decreased GCS score were risk factors for a poor prognosis.

## Data Availability

The raw data supporting the conclusions of this article will be made available by the authors, without undue reservation.
