# Supplementary material for: Elevated NLR and PCT levels and reduced GCS score predict 90-day mortality in heatstroke: findings from a 13-year retrospective cohort study
Source: Front Med (Lausanne). 2025 Jun 27;12:1599592. doi: 10.3389/fmed.2025.1599592 (PMC12245858; doi:10.3389/fmed.2025.1599592)
Supplement: Supplementary file 1 [file Table_1.docx]

**Supplementary Tables for**

**An elevated sustained neutrophil-lymphocyte ratio (NLR) was associated with 90-day case fatality in severe heat stroke: A 13-year retrospective cohort study**

**Table S1 Dynamic changes of the white blood cell counts in the survivor and non-survivor patients with heatstroke.**

|  | Overall | Survivor | Non-survivor | P value |
| --- | --- | --- | --- | --- |
| N | 232 | 200 | 32 |  |
| WBC |  |  |  |  |
| Admission | 11.15 [8.37, 14.48] | 11.03 [8.39, 14.36] | 11.67 [8.19, 16.00] | 0.622 |
| 24h | 9.02 [6.93, 12.14] | 8.80 [6.77, 11.59] | 9.70 [8.38, 13.73] | 0.064 |
| 48h | 8.13 [6.38, 10.75] | 7.97 [6.37, 10.06] | 10.91 [7.32, 13.75] | 0.005 |
| 72h | 7.14 [5.76, 9.54] | 6.98 [5.76, 8.93] | 9.69 [5.76, 12.54] | 0.056 |
| 5d | 6.99 [5.62, 9.83] | 6.92 [5.59, 9.46] | 8.52 [5.83, 12.08] | 0.252 |
| 7d | 8.40 [6.41, 11.98] | 7.81 [6.25, 10.09] | 14.06 [11.39, 17.92] | <0.001 |
| 14d | 8.48 [6.99, 12.56] | 8.19 [6.92, 10.26] | 18.88 [10.97, 19.19] | <0.001 |
| Neutrophil |  |  |  |  |
| Admission | 8.85 [6.34, 12.18] | 8.78 [6.35, 11.91] | 9.97 [6.38, 13.89] | 0.35 |
| 24h | 7.11 [4.99, 10.23] | 6.72 [4.80, 9.54] | 8.71 [7.46, 12.83] | <0.001 |
| 48h | 6.14 [4.34, 8.65] | 5.91 [4.13, 7.85] | 10.14 [6.75, 12.92] | <0.001 |
| 72h | 5.12 [3.95, 7.58] | 4.84 [3.95, 7.13] | 8.84 [4.95, 11.80] | 0.005 |
| 5d | 4.91 [3.50, 7.56] | 4.77 [3.47, 7.15] | 6.47 [3.64, 10.58] | 0.064 |
| 7d | 6.02 [4.28, 10.20] | 5.12 [4.10, 8.00] | 11.74 [8.76, 14.34] | <0.001 |
| 14d | 6.12 [4.49, 9.89] | 5.50 [4.09, 7.60] | 14.78 [9.35, 17.32] | <0.001 |
| Lymphocyte |  |  |  |  |
| Admission | 1.03 [0.56, 1.84] | 1.08 [0.62, 1.81] | 0.54 [0.29, 2.34] | 0.045 |
| 24h | 1.26 [0.70, 1.72] | 1.31 [0.96, 1.83] | 0.36 [0.28, 0.47] | <0.001 |
| 48h | 1.23 [0.62, 1.83] | 1.34 [0.86, 1.91] | 0.34 [0.22, 0.43] | <0.001 |
| 72h | 1.11 [0.65, 1.58] | 1.29 [0.74, 1.76] | 0.37 [0.23, 0.55] | <0.001 |
| 5d | 1.22 [0.71, 1.75] | 1.33 [0.93, 1.81] | 0.57 [0.25, 0.84] | <0.001 |
| 7d | 1.26 [0.82, 1.90] | 1.32 [0.86, 2.03] | 0.99 [0.50, 1.38] | 0.02 |
| 14d | 1.23 [0.85, 2.06] | 1.46 [0.95, 2.08] | 0.70 [0.28, 1.51] | 0.015 |
| NLR |  |  |  |  |
| Admission | 9.02 [4.21, 17.72] | 8.91 [4.06, 16.40] | 13.08 [5.09, 30.32] | 0.084 |
| 24h | 6.11 [3.23, 12.00] | 5.21 [3.00, 9.23] | 27.92 [19.80, 43.67] | <0.001 |
| 48h | 5.20 [2.81, 13.35] | 4.28 [2.54, 9.61] | 28.32 [20.76, 43.52] | <0.001 |
| 72h | 4.59 [2.81, 11.06] | 4.08 [2.52, 8.08] | 19.89 [11.66, 32.02] | <0.001 |
| 5d | 4.18 [2.41, 9.97] | 3.39 [2.30, 7.25] | 11.71 [6.39, 22.77] | <0.001 |
| 7d | 5.00 [2.77, 10.92] | 3.74 [2.48, 7.75] | 13.26 [7.75, 23.18] | <0.001 |
| 14d | 4.59 [2.74, 9.79] | 4.19 [2.31, 6.40] | 23.09 [9.79, 38.96] | <0.001 |

**Table S2 Comparison of the AUC of NLR for 90-day mortality at different time points.**

|  | **Threshold** | **Specificity** | **Sensitivity** | **Youden** | **95% CI** |
| --- | --- | --- | --- | --- | --- |
| Ad | 19.629 | 0.829 | 0.452 | 0.281 | 0.469-0.723 |
| 24h | 11.981 | 0.846 | 0.909 | 0.755 | 0.885-0.970 |
| 48h | 13.291 | 0.853 | 0.933 | 0.786 | 0.901-0.970 |
| 72h | 9.189 | 0.786 | 0.893 | 0.679 | 0.837-0.948 |
| 5d | 5.844 | 0.698 | 0.880 | 0.578 | 0.728-0.916 |
| 7d | 8.172 | 0.782 | 0.750 | 0.532 | 0.710-0.912 |
| 14d | 6.418 | 0.750 | 1.000 | 0.75 | 0.845-0.998 |

**Table S3 Comparison of the outcome between the heatstroke patients in NLR low group and NLR high group**

|  | **Overall** | **NLR =< 11** | **NLR > 11** | **P value** |
| --- | --- | --- | --- | --- |
| N | 225 | 158 | 67 |  |
| ICU time (d) | 5.00 [3.00, 10.25] | 4.00 [3.00, 7.00] | 9.00 [6.00, 15.50] | <0.001 |
| 90-day fatality |  |  |  |  |
| Survivor (%) | 195 (86.7) | 155 (98.1) | 40 (59.7) | <0.001 |
| Non-survivor (%) | 30 (13.3) | 3 (1.9) | 27 (40.3) |  |

**Table S4 Comparison of the characteristics between the Survivor and Non-survivor in NLR high group**

|  | **Overall** | **Survivor** | **Non-survivor** | **P value** |
| --- | --- | --- | --- | --- |
| N | 67 | 40 | 27 |  |
| APACHE II | 9.50 [4.00, 17.25] | 5.00 [2.00, 10.00] | 19.00 [18.00, 20.00] | <0.001 |
| SOFA | 6.00 [3.00, 11.00] | 4.00 [2.00, 6.00] | 13.00 [11.00, 14.00] | <0.001 |
| GCS | 11.00 [6.00, 14.00] | 13.00 [10.00, 14.00] | 5.00 [4.00, 6.00] | <0.001 |
| PLT | 49.00 [31.00, 63.00] | 54.50 [30.75, 70.00] | 49.00 [34.00, 57.00] | 0.531 |
| TBIL | 57.70 [40.20, 103.00] | 53.50 [37.85, 83.65] | 95.60 [49.37, 177.28] | 0.026 |
| ALT | 1074.00 [270.00, 2088.00] | 879.00 [226.50, 1727.50] | 1530.00 [664.25, 2397.25] | 0.147 |
| AST | 832.50 [242.50, 2693.75] | 634.00 [198.00, 1590.50] | 2545.00 [674.50, 5138.50] | 0.045 |
| BUN | 7.30 [4.80, 9.60] | 7.60 [4.90, 10.10] | 7.25 [4.80, 7.80] | 0.314 |
| SCR | 140.00 [93.00, 225.00] | 103.00 [76.00, 150.50] | 224.50 [196.25, 260.75] | <0.001 |
| CysC | 1.30 [0.88, 1.92] | 1.10 [0.84, 1.44] | 1.90 [1.36, 2.59] | 0.016 |
| CK | 3015.00 [1057.00, 7304.00] | 2252.00 [962.50, 5510.00] | 3344.00 [1373.75, 10300.50] | 0.226 |
| PT | 23.30 [16.60, 29.20] | 19.70 [16.25, 24.15] | 28.90 [24.75, 32.75] | 0.002 |
| INR | 2.12 [1.34, 2.88] | 1.67 [1.29, 2.20] | 2.76 [2.26, 3.26] | 0.002 |
| APTT | 47.10 [41.78, 62.90] | 43.20 [39.45, 50.00] | 67.20 [53.40, 75.70] | <0.001 |
| TT | 21.80 [17.10, 25.92] | 17.90 [16.90, 22.25] | 26.20 [22.20, 34.75] | 0.001 |
| Fib | 2.60 [2.02, 3.27] | 2.70 [2.25, 3.40] | 2.10 [1.80, 2.75] | 0.04 |
| D.D | 10.00 [6.56, 17.22] | 8.30 [3.36, 14.48] | 16.91 [10.00, 20.00] | 0.003 |
| Mb | 836.00 [237.00, 1000.00] | 338.00 [82.47, 1000.00] | 1000.00 [770.75, 1000.00] | 0.003 |
| CTNI | 260.00 [62.00, 688.85] | 70.00 [47.03, 460.00] | 620.40 [278.58, 2860.00] | <0.001 |
| PCT | 3.50 [1.81, 6.47] | 2.16 [1.65, 4.03] | 5.10 [3.76, 12.96] | 0.001 |
| CRP | 4.85 [3.30, 13.08] | 10.98 [5.13, 26.70] | 3.30 [2.94, 3.92] | 0.008 |

ALT: alanine transaminase; APACHE-Ⅱ: Acute Physiology and Chronic Health Evaluation-Ⅱ; APTT: activated partial thromboplasting time; AST: aspertate aminotransferase; BUN: blood urea nitrogen; CK: creatine kinase; CKMB: creatine kinase-myocardial band; CRP: C-reactive protein; CTNI: cardiac troponin I; CysC: cystatin C; D.D: d-dimer; Fib: fibrinogen; GCS: Glasgow coma scale; INR: international normalized ratio; Mb: myoglobin; PCT: procalcitonin; PLT: Platelet; PT: prothrombin time; SCR: serum creatinine; SOFA: sequential organ failure assessment; TBIL: total bilirubin; TT: thrombin time.
